# 3D Phase Field Modeling of Multi-Dendrites Evolution in Solidification and Validation by Synchrotron X-ray Tomography

**DOI:** 10.3390/ma14030520

**Published:** 2021-01-21

**Authors:** Shuo Wang, Zhipeng Guo, Jinwu Kang, Meishuai Zou, Xiaodong Li, Ang Zhang, Wenjia Du, Wei Zhang, Tung Lik Lee, Shoumei Xiong, Jiawei Mi

**Affiliations:** 1 School of Materials Science and Engineering, Beijing Institute of Technology, Beijing 100081, China; wangshuo@bit.edu.cn (S.W.); zoums@bit.edu.cn (M.Z.); bitlxd@bit.edu.cn (X.L.); 2 Institute for Aero Engine, Tsinghua University, Beijing 100081, China; 3 School of Materials Science and Engineering, Tsinghua University, Beijing 100084, China; Kangjw@mail.tsinghua.edu.cn (J.K.); smxiong@tsinghua.edu.cn (S.X.); 4 Key Laboratory for Advanced Materials Processing Technology, Ministry of Education, Beijing 100084, China; 5 College of Materials Science and Engineering, Chongqing University, Chongqing 400044, China; angzhang@cqu.edu.cn; 6 Department of Engineering, University of Hull, Cottingham Road, Hull HU6 7RX, UK; wenjiadumaterials@gmail.com (W.D.); w.zhang@supreium.com (W.Z.); 7 ISIS Neutron and Muon Source, STFC Rutherford Appleton Laboratory, Harwell Campus, Didcot, Oxfordshire OX11 0QX, UK; TUNG-LIK.LEE@STFC.AC.UK

**Keywords:** phase field modeling, dendrite evolution, solidification, synchrotron X-ray tomography

## Abstract

In this paper, the dynamics of multi-dendrite concurrent growth and coarsening of an Al-15 *wt*.% Cu alloy was studied using a highly computationally efficient 3D phase field model and real-time synchrotron X-ray micro-tomography. High fidelity multi-dendrite simulations were achieved and the results were compared directly with the time-evolved tomography datasets to quantify the relative importance of multi-dendritic growth and coarsening. Coarsening mechanisms under different solidification conditions were further elucidated. The dominant coarsening mechanisms change from small arm melting and interdendritic groove advancement to coalescence when the solid volume fraction approaches ~0.70. Both tomography experiments and phase field simulations indicated that multi-dendrite coarsening obeys the classical Lifshitz–Slyozov–Wagner theory $R^{n}-R_{0}^{n}{\;=\;k}_{c}\left({t-t}_{0}\right)$, but with a higher constant of *n* = 4.3.

## 1. Introduction

In metal castings, dendritic microstructures are the key microstructural features often formed in the solidification processes, determining, to a large extent, the mechanical properties of the finished cast products. In the solidification process, the growth of solid dendrites is always accompanied by coarsening. To reduce the free energy of the system, the coarsening process always occurs spontaneously by decreasing the overall curvature of the dendrites and the solid/liquid interface area [1,2]. Driven by the chemical potential, solute tends to diffuse from the area of lower curvature to that of higher curvature, a process that is governed by the Gibbs-Thomson equation:

(1) $$c_{curv}\left(T\right){\;=\;c}_{flat}\left(T\right)+\frac{\Gamma }{m}\kappa$$

where *c_curv_*(*T*), *c_flat_*(*T*) are solute concentrations at a curved and flat interface, respectively. Γ is the Gibbs-Thomson coefficient, *m* is the liquidus slope in a phase diagram, and κ is the curvature. Equation (1) clearly indicates that a higher curvature leads to lower concentration of solute. The diffusion of solute, driven by the uneven distribution of curvature, will normally result in (1) the remelting of small dendrite arms, (2) interdendritic groove advancement, and (3) coalescence between neighboring arms [3,4,5,6,7], as reported by many researchers in the past.

Extensive studies have been performed to investigate the coarsening behavior during dendrite growth. Much attention was focused on the studies of solid-liquid interface dynamics, i.e., interface motion [3,8], curvature distribution and transition [9,10], and subsequent solute diffusion. One of the key parameters used to describe the coarsening behavior is the characteristic length *R*, and it is the ratio between the volume of solid (*V_s_*) and the solid/liquid interface area (*S*). Hence, *R* = *V_s_*/*S*. In some cases, *R* = *V*_0_/S is used, where *V*_0_ is the entire volume of the sample. The two definitions only vary by a factor, *f_s_* (the volume fraction of solid), and it is a constant during dendritic coarsening in an isothermal condition. In the classic Lifshitz-Slyozov-Wagner (LSW) theory, *R* is mainly related to coarsening time *t* via [11]

(2) $$R^{n}{-R}_{0}^{n}{\;=\;k}_{c}\left({t\;-\;t}_{0}\right)$$

where *R*_0_ corresponds to the reference time *t*_0_ and *k_c_* is the rate constant that depends on the thermo-physical parameters of the alloy system. Extensive studies were carried out in solid-solid, semi-solid, and liquid-solid phase transformation processes to investigate the validity of this equation. However, most of the early studies were performed by characterizing the microstructures of the quenched samples [5,9], rather than “watching” the evolution of microstructures in real time during solidification. Hence, the time-evolved dynamic information was missing.

To obtain real-time information concerning dendrite growth and coarsening, in situ synchrotron X-ray imaging and/or tomography experiments were used by a number of researchers [7]. The time-evolved dendrite growth and coarsening data were used to find the exponent *n* of Equation (2). For example, Terzi et al. revealed that, depending on the local solidification conditions, in the coarsening process of Al-10%Cu alloy dendrites, the value of *n* was in the range of 3–5 [7]. However the value predicted by the LSW theory is 3, which has been widely accepted and used in the field of solidification research, especially for predicting dendrite coarsening behaviors [12]. Apparently, a higher *n* (such as those revealed by the in situ X-ray tomography experiments) or a lower *k_c_* indicate a slower coarsening rate during the growth of dendrites. Hence, the use of *n* = 3 (as predicted by the LSW theory and commonly used by previous research) could overestimate the coarsening rate of dendrite growth, resulting in errors in estimating other important microstructural features, for example, the secondary arm spacing of a microstructure.

In the 2010s, with the wide availability of synchrotron X-ray tomography facilities, real-time studies of dendritic morphology evolution in 3D and 4D (when time is included) were conducted by a number of researchers [3,7,8]. However, compared to the fast advances in 3D tomography experimental studies in the past ten years or so, the development of corresponding efficient modeling techniques that can accurately simulate the complex interface morphology evolution in 3D for multiple dendritic structures has been left far behind. The phase field method has been long accepted as the de facto technique for modeling complex interface problems with high fidelity. However, phase field modelling of multi-dendrite growth and coarsening in 3D needs an exceptionally efficient computing scheme. In this aspect, a number of numerical methods have been developed recently [13,14,15,16]. Among them, the adaptive mesh refinement has been proved to be one of the most computationally efficient methods, which has been used in the simulation of a single dendrite growth [13,14], multi-dendrite growth [17], and interface instability [18]. However, even with the adaptive mesh refinement, 3D phase field simulation of dendrite coarsening is still a computationally challenging task. In the case of simulating multi-dendrites in a solidification condition that is meaningful to industrial practice, for example in a large 3D domain that contains a few tens of dendrites and subject to a much longer solidification time, highly efficient parallel computing schemes need to be adopted. In this research, we combined the parallel computing technique with the adaptive mesh refinement method and implemented a parallel adaptive mesh refinement algorithm (named as Para-AMR algorithm hereafter). Numerical tests showed that by employing such Para-AMR algorithm, the computational efficiency was increased by approximately three orders of magnitude [19].

Using such highly efficient 3D phase field model, we were able to simulate multi-dendrite growth and coarsening behaviors and compared the simulated results directly with the experimental results obtained from synchrotron X-ray tomography. The studies were focused on two aspects. The first was to fit the key parameters in Equation (2), including the exponent *n* and the rate constant *k_c_* using both experimental and simulation results. The obtained results were compared with those predicted by the classic LSW theory and those from the open literature. The second was to elucidate the different coarsening mechanisms that govern dendrite growth and coarsening and their relevant importance at different stages of solidification. Using the two state-of-the-art techniques, i.e., synchrotron X-ray ultrafast tomography and a highly computationally efficient 3D phase field model, the kinetics of multi-dendrite growth and coarsening were studied in a much more quantitative way. The relative importance of the different mechanisms of dendrite coarsening were further clarified.

## 2. Experiments

### 2.1. Alloy and Sample Preparation

An Al-15 *wt*.% Cu alloy was used for the experiment. The alloy was made by melting together pure Al (purity of 99.97%), and pure Cu (purity of 99.97%) with the designed weight ratio in a quartz beaker (47 mm inner diameter and 64 mm deep) using an electric resistance furnace. The alloy was held at ~730 °C for ~1 h to homogenize the melt. The alloy melt was then drawn uphill and poured into a quartz tube of 100 mm long with an inner diameter of 2.5 mm by using a small counter-gravity casting apparatus [17]. The negative pressure (~0.5 atmospheric pressure) was able to draw the liquid metal into the quartz tubes in a quiescent manner, avoiding any surface turbulence during the filling and minimizing any possible entrainment of air bubbles or oxide films into the cast bar. After solidification, the bar sample was taken out and inserted into a new quartz tube (3 mm inner diameter and 1 mm wall thickness) in preparation for the subsequent X-ray tomography experiments.

### 2.2. Real-Time Synchrotron X-ray Tomography

**Figure 1:**
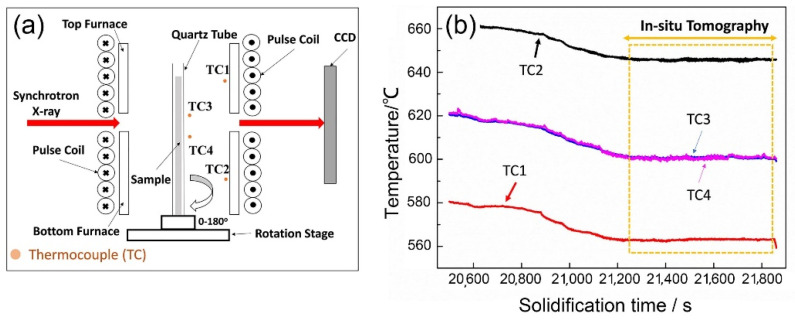
(**a**) A schematic illustration of the experimental setup. (**b**) The measured temperature profiles of TC1, TC2, TC3, and TC4 during the solidification experiments. The framed area is the period during which tomography scans were performed.

Real-time synchrotron X-ray tomography experiments were carried out at the Tomographic Microscopy and Coherent Radiology Experiments (TOMCAT) beamline of Swiss Light Source, Paul Scherrer Institute, Switzerland using a specially designed pulse electromagnetic field solidification apparatus [17]. Figure 1a shows the schematic illustration of the experimental setup. Two furnaces were used to heat the alloy sample. Four k-type thermocouples were positioned at the locations inside the furnaces and marked by TC1, TC2, TC3, and TC4 in Figure 1a. TC1 and TC2 are the temperature control points for the two furnaces. TC3 and TC4 are the points very close to the quartz tube and the distance between them was 10 mm. The two thermocouples were kept outside the quartz tube to avoid any entanglement of the thermocouple wires due to sample rotation in tomography scans. Temperature calibration was performed with one thermocouple inserted inside the quartz tube at the location between TC3 and TC4. The measurement showed that the difference between the measured temperatures inside and outside the quartz tube was just ~1 °C.

During experiments, the two furnaces were firstly heated up to ~700 °C to melt the alloy completely. The temperature of the top and bottom furnaces was then simultaneously decreased until the sample temperature (showed by the readings from TC3 and TC4) reached ~610 °C (Figure 1b). After this, the temperature was maintained, and tomography scans started. A polychromatic X-ray beam filtered to 50% power and a pco.edge 5.5 detector (PCO AG, Kelheim, Germany) with a 100 μm thick LAG:Ce scintillator were used. Sample-to-detector distance was set to 300 mm. A 10× magnification objective lens was used, resulting in a pixel size of 0.65 μm with a field of view of 2016 × 2016 pixels (equivalent to ~1.31 × 1.31 m^2^), which is able to contain sufficient number of dendrites for statistical analyses. Five hundred projections were acquired in an angular step of 0.36° over a 180° rotation, each with an exposure time of 7 ms. Hence, one tomography scan was completed in 3.5 s, and the skip time between two continuous scans was 10 s. The scans were taken continuously for about 6.7 min (see the framed region in Figure 1b).

### 2.3. Image Processing

**Figure 2:**
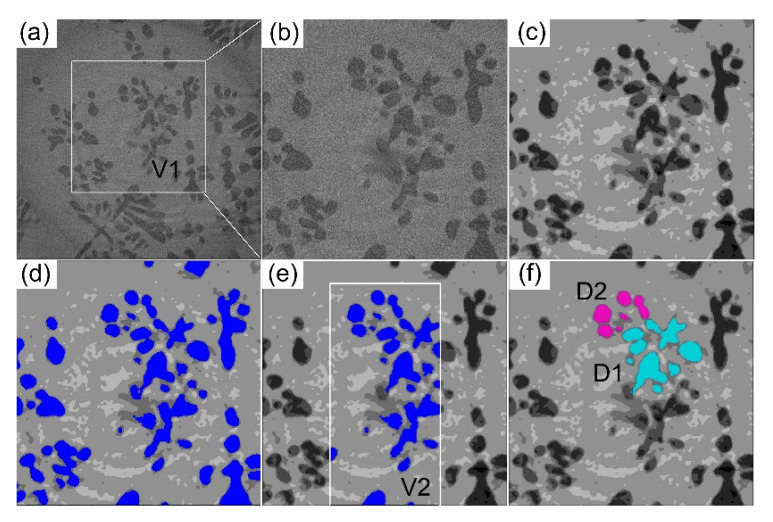
(**a**) A typical 2D projection selected from the synchrotron X-ray tomoscans containing multiple equiaxed dendrites. (**b**–**d**) The methods for processing the multiple dendrites from the raw images including cropping the subvolume V1, applying a 3D median filter, and thresholding. (**e**,**f**) The extractions of subvolume V2 (used for comparing with the results from phase filed simulation) and dendrites D1 and D2.

The 16-bit raw tomography datasets were firstly binned and converted into 8-bit image stacks using Matlab using PITRE developed by INFN Trieste [20] and then segmented and rendered using Avizo^®^ [21] to reveal the 3D dendrites, as shown in Figure 2. Multiple dendrites contained in the subvolumes (V1 and V2), and dendrites marked by D1 and D2 were selected and extracted for further analysis. Methods, including subvolume selection, 3D median filtering, thresholding, segmentation, and smoothing, were used to achieve better microstructure rendering [18].

## 3. Phase Field Model and Numerical Scheme

### 3.1. The 3D Phase Field Model

An isothermal 3D phase field model [19,22,23] was adopted in this research, and the governing equations for phase field and solute are:

(3) $$\begin{array}{c} \tau \frac{\partial \phi }{\partial t}=\nabla \cdot (W{\left(\vec{n}\right)}^{2}\nabla \phi )+\frac{\partial }{\partial x}({|\nabla \phi |}^{2}W\left(\vec{n}\right)\frac{\partial W\left(\vec{n}\right)}{\partial \phi_{x}})+\frac{\partial }{\partial y}({|\nabla \phi |}^{2}W\left(\vec{n}\right)\frac{\partial W\left(\vec{n}\right)}{\partial \phi_{y}})\\ +\frac{\partial }{\partial z}({|\nabla \phi |}^{2}W\left(\vec{n}\right)\frac{\partial W\left(\vec{n}\right)}{\partial \phi_{z}})+\phi (1-\phi^{2})-\lambda {\left(1-\phi^{2}\right)}^{2}(\theta +kU) \end{array}$$

(4) $$\left(\frac{1+k}{2}-\frac{1-k}{2}\phi \right)\frac{\partial U}{\partial t}=\nabla \cdot \left(D\frac{1-\phi }{2}\nabla U+\frac{W}{\sqrt{2}}\frac{{c/c}_{\infty }}{\left[1+k-(1-k)\phi \right]}\frac{\partial \phi }{\partial t}\frac{\nabla \phi }{\left|\nabla \phi \right|}\right)+\frac{1}{2}\left[1+\left(1-k\right)U\right]\frac{\partial \phi }{\partial t}$$

where *φ* is the phase field, *τ* is the relaxation time, $W(\vec{n})$ is the anisotropic width of the diffuse interface, $\vec{n}$ is unit vector in the normal direction, *k* is the partition coefficient for the solute, *D* is the solute diffusivity, and *λ* is the scaling parameter. *U* and *θ* (see Equations (5) and (6) below [16])

(5) $$U\;=\;\frac{\frac{{2c/c}_{\infty }}{1\;+k-(1-k)\phi }-1}{1-k}$$

(6) $$\theta \;=\;\frac{{\;T-T}_{m}{-mc}_{\infty }}{{\Delta T}_{0}}$$

are dimensionless solute concentration and temperature, respectively [19]. *c* is solute concentration, *c*_∞_ is the initial solute concentration, *m* is the liquidus slope in a phase diagram, *T*_m_ is the melting temperature, and Δ*T*_0_ is the equilibrium freezing temperature range according to *c*_∞_.

In this work, crystal anisotropy was introduced by taking $\tau =\tau_{0}A{\left(\vec{n}\right)}^{2}$, $W(\vec{n})=W_{0}A(\vec{n})$ and

(7) $$A\left(\vec{n}\right)=1+\varepsilon_{1}(\frac{\phi_{x}^{4}+\phi_{y}^{4}+\phi_{z}^{4}}{{|\phi |}^{4}}-3/5)+\varepsilon_{2}(3\frac{\phi_{x}^{4}+\phi_{y}^{4}+\phi_{z}^{4}}{{|\phi |}^{4}}+66\frac{\phi_{x}^{2}\phi_{y}^{2}\phi_{z}^{2}}{{|\phi |}^{6}}-17/7)$$

where *φ_x_* = ∂*φ* /∂*x*, *φ_y_* = ∂*φ* /∂*y*, and *φ_z_* = ∂*φ* /∂*z*. *ε_1_* and *ε_2_* are the weighting factors accounting for the magnitude of anisotropy strength in crystal directions (100) and (110), respectively. The length and time in Equations (3) and (4) were scaled by *W*_0_ = *λd*_0_/*a*_1_ and $\tau_{0}{\;=\;d}_{0}^{2}a_{2}\lambda^{3}{/Da}_{1}^{2}$ to make Equations (3) and (4) dimensionless. *d*_0_ = *Γ*/∆*T*_0_ is the chemical capillary length, *a*_1_ = 0.8839 and *a*_2_ = 0.6267 [22,23]. Derivation of the governing equations and section of parameters are detailed in [19,22,23]. A Para-AMR algorithm with adaptive mesh refinement and parallel computing ability was used to solve Equations (3) and (4). Detailed numerical schemes and implementation can be found in [19] and are not repeated here.

### 3.2. Numerical Experiments

**Figure 3:**
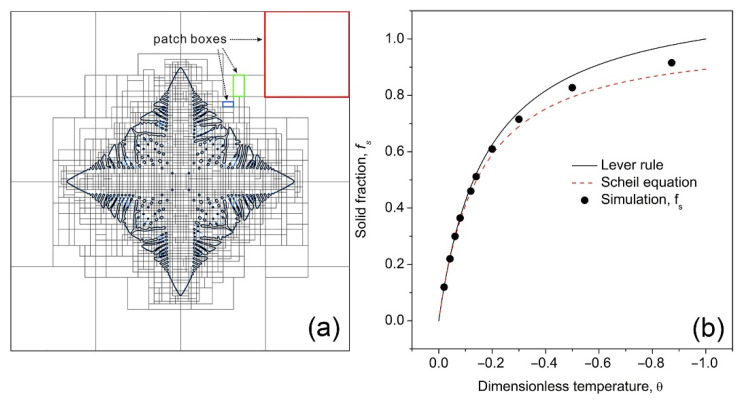
(**a**) An example of the adaptive mesh structure on the x-y plane generated using the block-structured adaptive mesh refinement algorithm. (**b**) The calculated volume fraction of solid, *f_s_* using the 3D phase field model and comparison with those calculated using the Lever rule and Scheil equation.

A single Al-15 *wt.*% Cu alloy dendrite was chosen for numerical experiments and the parameters used in the simulations were *k* = 0.15 [9], *λ* = 30, *ε_1_* =0.15, and *ε_2_* = 0. Figure 3a shows the typical adaptive mesh structure in the x-y plane in the middle of the simulation. A solid seed was firstly placed at the center of the domain, and it grew isothermally and gradually evolved into a dendrite. The simulation continued until the dendrite filled the whole domain with the solid volume fraction approaching a constant value. Figure 3b shows the “equilibrium” solid volume fraction (marked by the black dots) obtained from simulations at 10 dimensionless temperatures (*θ* = −0.02, −0.04, −0.06, −0.08, −0.12, −0.14, −0.2, −0.3, −0.5, and −0.9) and comparison with those calculated by using the Lever rule and Scheil equation:

(8) $$f_{s}^{lever}\;=\;\frac{-\theta }{k-\theta \left(1-k\right)}$$

(9) $$f_{s}^{scheik}\;=\;1-{\left[1-\theta \frac{1-k}{k}\right]}^{\frac{1}{k-1}}$$

The Lever rule assumed that a full thermodynamic equilibrium was reached with uniform solute composition in the liquid and solid phase in the alloy system. In the Scheil equation, solute diffusion in the solid phase was ignored. Figure 3b shows that the simulated solid volume fraction follows the same trend as those predicted by the Lever rule and Scheil equation, and are closer to the curve calculated by the Scheil equation, especially at lower temperatures. This was because in the current phase field modeling, solute diffusion in the solid phase was not considered. Hence, from a thermodynamic point of view, the calculated solid volume fraction from the phase field simulation agrees well with the classical theory. However, the phase field simulation is able to calculate and accurately describe the time-evolved morphology changes of multi-dendrites and their interactions.

## 4. Results

### 4.1. Dendrite Evolution Revealed by Synchrotron X-ray Tomography

**Figure 4:**
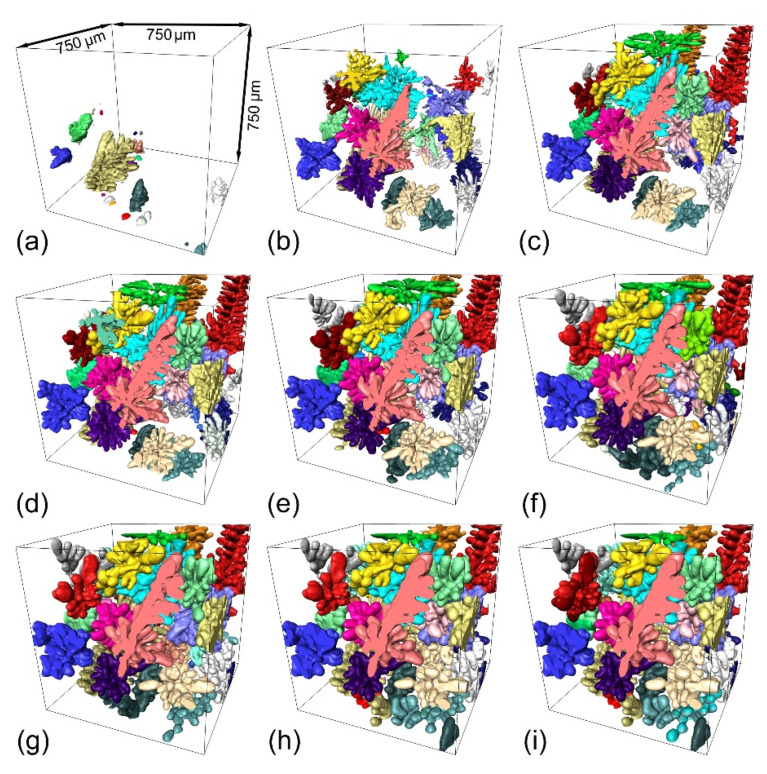
The growth and coarsening of Al-15 *wt*.% Cu alloy multiple dendrites at the solidification times of 10, 20, 30, 40, 60, 110, 160, 210, and 300 s as shown in (**a**–**i**) (see Video S1 for the growth dynamics).

Figure 4 shows the growth and coarsening of the multiple dendrites contained in subvolume V1 (715 × 715 × 715 µm^3^) in Figure 2a. A total of 30 dendrites were identified and found to nucleate initially at the lower region of the domain (Figure 4a). After first appearing in Figure 4a, the dendrites grew relatively fast and filled the majority of V1 in less than 30 s (Figure 4a–c). The dendrites also coarsened simultaneously as they grew. Coarsening occurred much faster at the beginning (comparing the coarsening behavior from Figure 4b–e to that from Figure 4e–i). Actually, the dendrite morphology did not change much after 210 s, i.e., from Figure 4h,i.

**Figure 5:**
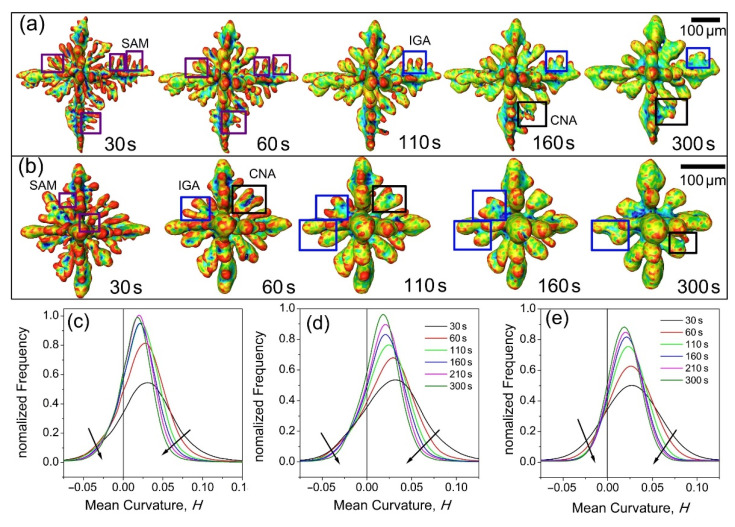
(**a**) The evolution of morphology and curvature for dendrite D1 and (**b**) those for D2. Different coarsening mechanisms were found at different stages of growth (marked with squares), and they are small arm melting (SAM), interdendritic groove advancement (IGA), and coalescence between neighboring arms (CNA), respectively. Video S2 and Video S3 show more clearly the dynamic evolution process. (**c**) The surface area (normalized frequency) as a function of curvature for all dendrites shown in Figure 4, and that for dendrite D1 (**d**) and D2 (**e**).

To better understand the mechanisms of growth and coarsening, two of the dendrites enclosed in V1 were extracted and are shown in Figure 5, demonstrating more clearly their morphology evolution from 60 s to 300 s. Small arm melting (SAM) was observed at an early stage of the coarsening of dendrites D1 and D2, as indicated by the areas framed in purple. Interdendritic groove advancement (IGA) and coalescence between neighboring arms (CNA) were both observed at D1 and D2 from 60 s to 300s, as shown in the marked areas with blue and black frames in Figure 5a,b. The driving forces for small arm melting and interdendritic groove advancement are rather simple and could be mostly attributed to the curvature effect according to Equation (1). The higher curvature of the small convex arms attracted neighboring solute diffusing towards it and remelting it [24]. The diffusing away of solute at a lower curvature area, such as the concave groove, made it easy to grow further. This is the mechanism for IGA [25]. The IGA is much slower, which is probably due to the long diffusion distance from roots to tips. The whole coarsening process can also be seen as the coalescence between neighboring arms (CNA) [25], which means that several coarsening mechanisms may operate simultaneously and shift with increased solidification. However, the CNA is rather complicated, and involves the attraction and connection of neighboring arms. The coalescence could be one of the effects due to Rayleigh instabilities, which has been studied by Aagesen et al. [8] using both X-radiation experiments and analytical methods. The distinction between solidification growth and coalescence is very difficult to make, as solidification and coarsening are occurring at the same time [4]. So, we just take the solidification and coarsening as a whole process. SAM usually occurred at an early stage in a short period of time. CNA was always found at a late stage (from 110s to 300s). IGA was found throughout the growing and coarsening of dendrites.

Statistical analyses of the surface area (normalized frequency) as a function of curvature from 30 to 300 s were performed for all dendrites in Figure 4, and for the dendrites, D1 and D2 as well. The results are shown in Figure 5c–e. Clearly, from 30 s to 110 s, the mean curvature distribution profiles changed from a relatively broader profile to a narrower profile and the peak of the profile shifted from a more positive curvature to a less positive curvature, indicating that the dendrites with a higher mean curvature evolved into a morphology with relatively lower mean curvatures. Such changes reflect that small dendrites with a more positive curvature were melted and disappeared due to SAM [3,4,5,26,27].

At the same time, the proportion of negative curvature decreased in the profile from 30 s to 300 s, as marked with black arrows, indicating that the regions containing negative curvatures decreased as well. The roots between two secondary arms are the typical area with negative curvatures, and the effect of IGA resulted in an increase in such areas [28]. In addition, the red arrows show the increase in negative curvature, especially in the profile from 110 s to 300 s. The negative curvatures increased and the surface area decreased when coalescence between neighboring arms occurred, and thus CNA can be attributed to the increasing of negative curvatures. From 210 s to 300 s, the change of the curvature profiles and their peak positions was very small, indicating that further coarsening was realized by smearing and smoothing of the solid-liquid interface. The surface area decreased from 210 s to 300 s as a result of CNA, as clearly demonstrated by Video S1 and Video S2. Both morphology and statistical analyses show that small arm melting is dominant in the early stage of coarsening, while coalescence is the main mechanism at the later stage of coarsening.

### 4.2. Phase Field Modeling

#### 4.2.1. Determining the Locations and Orientations of Dendrite Seeds

**Figure 6:**
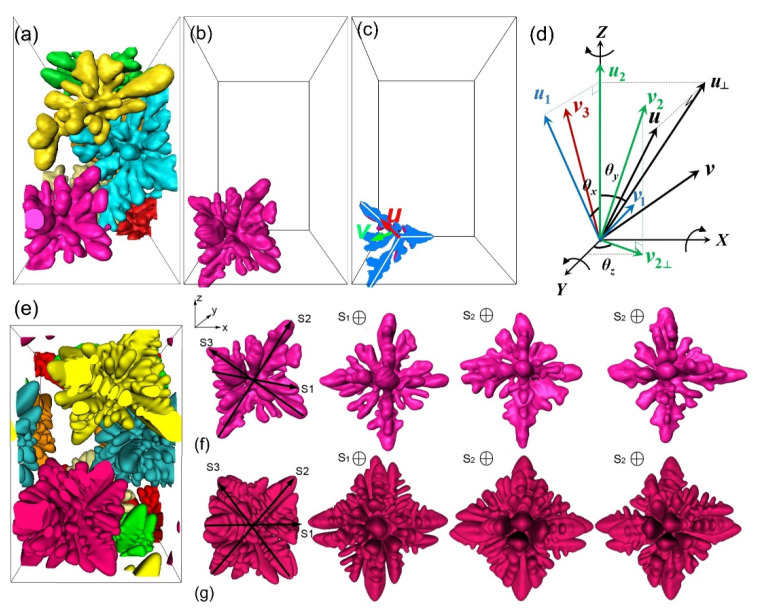
(**a**) The multiple dendrites (at solidification time of 110 s) segmented from V2 of Figure 2e. (**b**) A single turquoise-colored dendrite selected from (**a**). (**c**) Orthogonal plane section through the primary arm and secondary arm of the turquoise dendrite, and the unit vectors that constitute the local co-ordinate (*u*, *u*’, *v*’) of the dendrite. (**d**) The global co-ordinate (*X, Y, Z*), and how the dendrite’s local co-ordinate information was mapped onto the global system. (**e**) Simulated multiple dendrites corresponding to the experimental dendrites shown in (**a**), (**f**,**g**) comparison between a single dendrite evolution from an experiment (**f** row) and the corresponding simulated dendrite (**g** row), S_1_–S_3_ are the three orthogonal planes of α-Al.

In order to simulate the growth and coarsening of multiple dendrites in conditions as close as possible to the experimental conditions, the locations and orientations of the multiple dendrite seeds to be planted into a 3D computational domain need to be determined from experiments. We used the following procedure to achieve this. From the tomography datasets shown in subvolume V2 of Figure 2e, we firstly segmented all dendrites in that subvolume (Figure 6a) and then worked on each individual dendrite to retrieve its local co-ordinates and growing directions of its dendritic arms in 3D space. Taking the turquoise-colored dendrite shown in Figure 6b, for example, three orthogonal planes were used to section the dendrite along its primary arms and secondary arms, as shown in Figure 6c. For the primary arm of the α-Al dendrite to be orthogonal, once a primary arm was located in a certain direction in the reference co-ordinate axis, the others would be located in a perpendicular plane without certain orientations. Thus, the orientation of one secondary arm was essential to finally determine the dendritic orientation. So, directions of the unit vectors for the primary arm and the secondary arm were determined and considered as the local co-ordinate (*u*, *v*) for this particular dendrite. Finally, the angles of the local co-ordinate axis (*u*, *v*) with respect to a predefined global reference co-ordinate axis (*X-Y-Z* in Figure 6d) can be retrieved and rotation matrices as below were created to map the local co-ordinate angle information onto the global reference co-ordinate system with a rotation order of *Z*, *X*, Y, positive for anti-clockwise rotation and negative for clockwise rotation. Table 1 gives a detailed formula for the mapping operation.

$$R_{x}(\theta )\;=\;\left[\begin{array}{ccc} 1 & 0 & 0\\ 0 & \cos\theta & -\sin\theta \\ 0 & \sin\theta & \cos\theta \end{array}\right]{\;R}_{y}(\theta )\;=\;\left[\begin{array}{ccc} \cos\theta & 0 & \sin\theta \\ 0 & 1 & 0\\ -\sin\theta & 0 & \cos\theta \end{array}\right]{\;R}_{z}(\theta )\;=\;\left[\begin{array}{ccc} \cos\theta & -\sin\theta & 0\\ \sin\theta & \cos\theta & 0\\ 0 & 0 & 1 \end{array}\right]$$

#### 4.2.2. Computational Domain, Parameters Used, and Modeling Results

For quantitative comparisons between the simulated dendrites and those from tomography experiments in V2 of Figure 2e (see Video S4), a computational domain of 400 × 600 × 800 (dimensionless, equivalent to a uniform orthogonal grid of 500 × 750 × 1000 = 1.07 × 10^9^ cells) was used. The mesh structure used in the simulation has five levels. The coarsest grid size was *dx_max_* = 12.8, while the finest was *dx_min_* = 0.8. In addition, the mesh size in each grid level was set to be half of that of the nearest coarser grids. The time step for the simulation was *dt* = 0.8 × *dx_min_*^2^/(6*λa*_2_). Seven seeds (their locations and orientations were retrieved from the tomography data) were planted simultaneously (i.e., the order of nucleation was not considered when planting the seeds) into the domain and then grew simultaneously. The temperature of the system was set as *θ* = −0.031, so the calculated initial solid volume fraction (according to the Lever rule) was 0.17, very similar to that observed from the experiment. A low undercooling of 0.031Δ*T*_0_ and relatively larger scaling parameter (*λ* = 300) were used to balance the growth and subsequent coarsening of the simulated dendrites. The other parameters used are listed in Table 2. Periodic boundary conditions were employed in the simulations. Numerical tests indicated that, using 192 cores of the “Landau” computing cluster hosted at Tsinghua University, an equilibrium condition (i.e., until the volume fraction of solid remained constant) was achieved in an approximately 7 × 10^5^ time step calculation (~20 h computing). In total, about ten million time steps were computed in the simulation.

Figure 6d shows one snapshot (at solidification time of 210 s) of the simulated multiple dendrites, corresponding to the experimental dendrites shown in Figure 6a. Meanwhile, Figure 6f,g compare the dendrite morphology in different view directions (S1, S2, and S3) between the experimental and simulated dendrites. Clearly, the simulated and experimental dendrites exhibit highly similar characteristics of size, hierarchical dendrite branches, orientation, and surface morphology.

**Figure 7:**
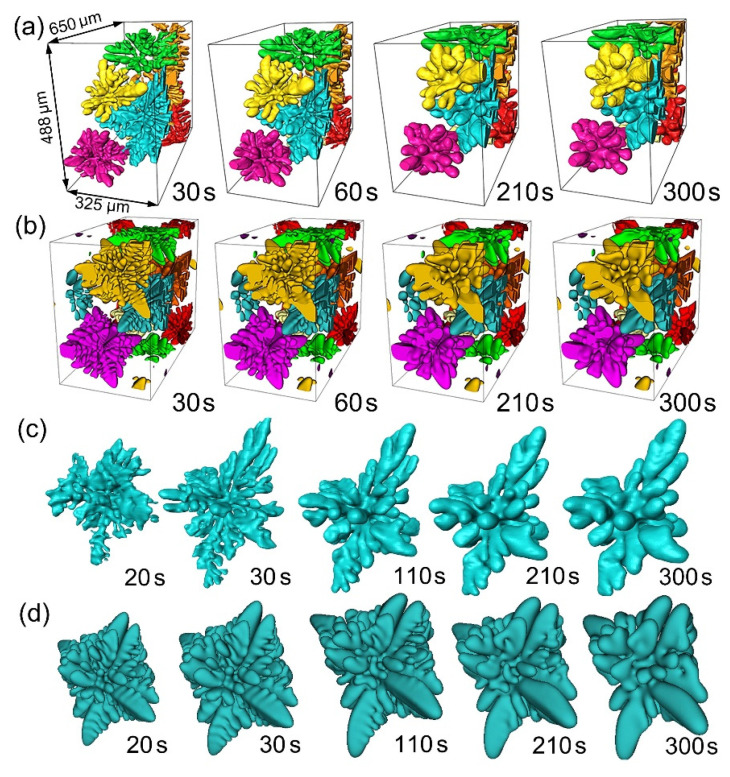
(**a**) Multi-dendrite (those from V2 of Figure 2e) growth and coarsening at the solidification times of 30, 60, 210, and 300 s and (**b**) the corresponding phase field simulated ones. (**c**) A single turquoise-colored dendrite selected from Figure 7a, and (**d**) the corresponding phase field simulation.

Figure 7 shows more snapshots of the experimental and simulated multi-dendrites (Figure 7a,b) at different solidification times (30, 60, 210, and 300 s), while Figure 7c,d show more clearly the single turquoise-colored dendrite. Video S4 and Video S5 provide more vivid dynamic information for the experimental and simulated multiple dendrites. It can be seen that the simulated and experimental dendrites exhibit highly similar characteristics of size, hierarchical dendrite branches, orientation, and surface morphology, demonstrating that the phase field is able to provide an efficient modeling of 3D multiple dendrite evolution with time.

## 5. Discussions

### 5.1. Statistical Analyses on the Simulated and Experimental Dendrites

**Figure 8:**
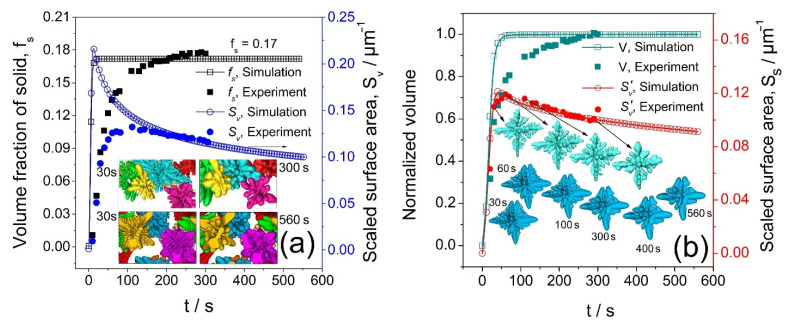
(**a**) The solid fraction and specific surface area of the multi-dendrites (those from V2 of Figure 2e) as a function of time and those from the phase field modeling. (**b**) The normalized volume (*V*/*V*_max_, *V*_max_ is the maximum volume of the dendrite) and scaled surface area *S*_S_ (*S*/*V*) of dendrite D1 in Figure 2f as a function of time and those from the phase field modeling.

To further analyze the accuracy and robustness of the phase field modeling, a systematic statistical analysis was done using the rich datasets obtained from modeling and tomography experiments. For Volume 2 of Figure 2e, the solid volume fraction (*f_s_*) and the normalized L-S interface (the dendrite surface area, i.e., *S_v_* = *S*/*V*_0_, *V*_0_ is the total volume of the domain) were retrieved from both simulated and tomography datasets. The time-evolved *f_s_* and *S_v_* were plotted in Figure 8. For dendrite D1, *f_s_* becomes the normalized volume of the dendrite, and *S_v_* becomes *S_s_*, defined as solid-liquid interface area per unit volume enclosed by the interface of a single dendrite [29].

Figure 8a shows the time-evolved *f*_s_ and *S_v_* for the multi-dendrites in V2 of Figure 2e and those from the phase field simulation. The *f*_s_ from the tomography experiment showed a rapid increase at the initial stage of solidification, and then approached a constant value of ~0.17 after *t* = 200 s. However, *S_v_* from the tomography experiment decreased gradually after reaching its maximum at ~20 s. In the simulation, it took a much shorter time for the *f*_s_ to reach its equilibrium value, i.e., 0.17. Despite the initial difference, the simulated and experimental *f_s_* and *S_v_* reached approximately the same value at around *t* = 200 s, and then maintained the same value.

The initial difference between the experiment and modeling was that, in the experiment, the temperature was decreased gradually and the dendrites were nucleated at different times. The initial driving force for dendrite growth was small because of the gradual decrease in temperature. Meanwhile, in the simulation, the whole domain was set to be isothermal at once and all dendrites grew simultaneously from the beginning of the simulation. Hence, there was more driving force to grow multi-dendrites from the very beginning for the planted dendrites. However, this difference decreased after the normalized surface area approached a constant value.

Figure 8b shows the normalized volume and scaled surface area of one typical dendrite (D1 of Figure 2f) and those from the simulation. Similar to those in Figure 8a, there is a mismatch for the normalized volume between the experiment and simulation at the initial solidification stage. However, the two datasets agreed well at *t* = 200 s. It is important to see that this initial difference does not affect the results after 200 s, i.e., when much of the domain was filled with dendrites. This gives us solid confidence that, although no tomography scan was acquired after 300 s due to the storage issue, we can use the simulated data to study the coarsening behavior on a much longer time scale.

### 5.2. Coarsening Mechanisms in Different Thermal Conditions

As discussed, during coarsening the curvature evolves, aiming at decreasing the overall interface area. It is reasonable to use simulation to investigate the coarsening during solidification according to the comparison of the simulation and experimental results in Section 5.1. To investigate the curvature transition, a simulation was performed using the same configuration in Table 1 with ten dendrites seeded randomly in a domain with a size of 512 × 512 × 512. A hierarchical five-level grid structure was constructed and the grid size on the top level was set to *dx* = 0.8, and the time step employed in this study was *dt* = 0.8 × *dx*^2^/6D. The period boundary conditions were applied, and the scaling parameter, i.e., *λ* = 30, was employed. Different isothermal temperatures, i.e., *θ* = −0.05, −0.10, −0.12, −0.20, −0.30, −0.50, −0.70, −0.90, were used to investigate the change of coarsening mechanisms in different thermal conditions. To get fully coarsened cases, 8 × 10^4^ *dt* was adopted.

**Figure 9:**
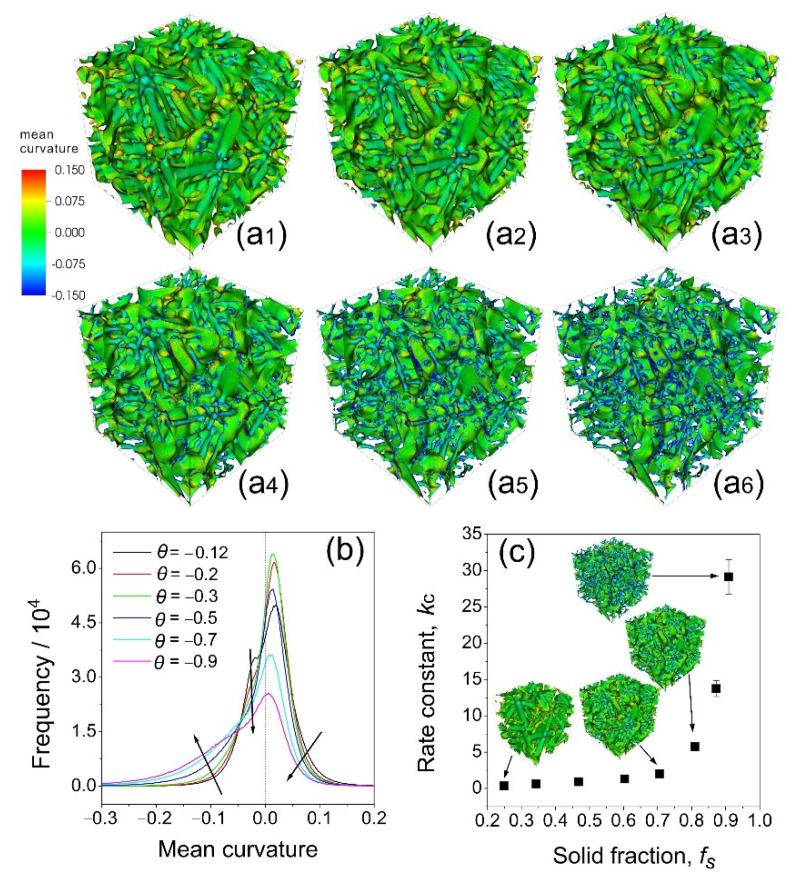
(**a_1_**–**a_6_**,**b**) The snapshots and the distribution of mean curvature changes of the simulated domain at different isothermal temperatures at 8 × 10^4^ *dt*, respectively. (**a_1_**–**a_6_**) Snapshots of simulated domain at *θ* = −0.12, −0.20, −0.30, −0.50, −0.70, and −0.90. (**c**) The comparison between simulation and fitted results for different simulation cases.

Figure 9a_1_–a_6_ shows the snapshots of the mean curvature changes under different isothermal temperatures (i.e., *θ* = −0.12, −0.20, −0.30, −0.50, −0.70, −0.90 from Figure 9a_1_–a_6_). For Figure 9a_1_–a_3_, the mean curvatures of dendrites were similar except for a small increase in negative curvature. However, when the isothermal temperature decreased to −0.50 (as in Figure 9a_4_), the negative curvature increased dramatically and it increased further as the isothermal temperature decreased further. Meanwhile, the positive curvature in Figure 9a_4_–a_6_ decreased as the isothermal temperature decreased. The mean curvature distribution under different isothermal temperatures in Figure 9b showed a clear increase in the negative curvatures from cases of *θ* = −0.12 to those of *θ* = −0.90 and the decrease in total surface area decreased in cases of *θ* = −0.30 to *θ* = −0.90.

To study the transition of the coarsening mechanisms more quantitatively, statistical analyses of the counted surface area as a function of the mean curvature were performed. Figure 9b shows that the peak value of the distribution first increased as the temperature decreased from *θ* = −0.12 to *θ* = −0.30 and then decreased from *θ* = −0.30 to *θ* = −0.90. This clearly indicated that there is a change at *θ* = −0.30 for the coarsening mechanism, and when *θ* < −0.30, the coalescence became dominant. The rapid reduction in the surface areas with zero curvature, and the increase in those with negative curvatures, provide further solid evidence. When the temperature reached −0.30, the equilibrium solid fraction was about 0.72 (by simulation), 0.74 (by the Lever rule), and 0.69 (by the Scheil equation). It is not difficult to imagine that, at this level of solid volume fraction, the interfaces between neighboring arms became so close that coalescence occurred.

Previous studies [11,29] showed that the rate constant *k_c_* was highly dependent on the volume fraction of the coarsening phase. Hardy and Voorhees [29] used Sn-rich and Pb-rich particles with the solid fraction in the range *f*_s_ = 0.6~0.9. The coarsening rate, *k*_c_, was shown to increase abruptly as *f*_s_ approached unity. Figure 9c shows that the rate constant *k_c_* increases exponentially as the solid fraction increases, and when the solid fraction *f*_s_ > ~0.7, the coarsening rate increased abruptly, clearly indicating the change of mechanism for coarsening under a big solid fraction, i.e., the occurrence of interface attachment between neighboring arms coalescence. This is further confirmed by the snapshots of mean curvature changes shown in Figure 9a_1_–a_6_, and the distributions of the mean curvatures move into the negative region, as shown in Figure 9b, when *θ* < −0.30, i.e., *f*_s_ > ~0.7. Basically, at this stage, the coalescence between neighboring arms became a dominant phenomenon.

### 5.3. Comparison with Classical LSW Theory for Dendritic Coarsening

In the classical LSW theory, i.e., $R^{n}{-R}_{0}^{n}{\;=\;k}_{c}\left({t-t}_{0}\right),$ *R* is the average radius (for spherical particles) of particles, *R*_0_ is the reference radius at time *t*_0_, and *k*_c_ is a rate constant that is dependent on diffusivity and thermo-physical parameters. In the past, when LSW theory was applied to the coarsening of dendrites during solidification, *n* = 3 had been used arbitrarily [2]. Recently, using numerous dendritic coarsening datasets obtained by real-time X-ray tomography, some researchers have again tested the suitability of LSW theory for dendrite coarsening, and found that the *n* is in the range of 2.5 to 7.5 [5,30,31,32,33].

**Figure 10:**
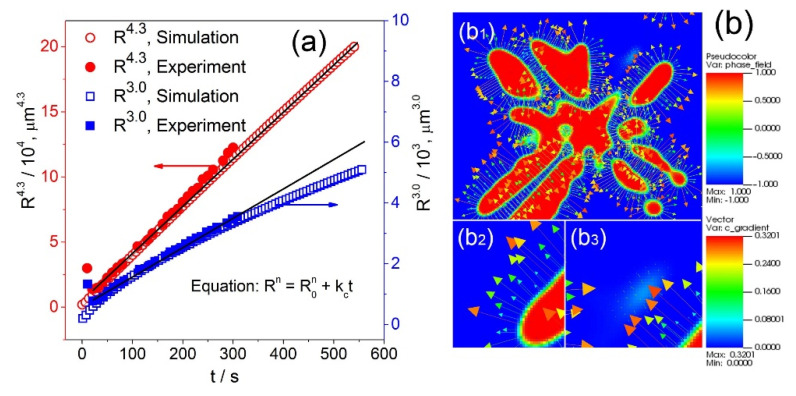
(**a**) The fitted results using *n* = 3.0 and *n* = 4.3 according to $R^{n}\;-{\;R}_{0}^{n}{\;=\;k}_{c}\left(t\;-{\;t}_{0}\right),$ (**b**) the gradient of solute concentration in the cross section of a phase field simulated dendrite.

In our work, we define *R* = *V_s_*/*S*, and plot *R*^4.3^ and *R*^3.0^ as a function of time, and the results are shown in Figure 10a. Despite a very small deviation at *t* < 30 s, the *R*^4.3^ case showed an excellent agreement between the simulated and experimental datasets. However, for the *R*^3.0^ case, the difference between the simulated and the experimental results became bigger and bigger as the dendrite coarsening continued.

The simulation results clearly show that dendrite coarsening indeed follows the LSW theory with *n* = 4.3 (not *n* = 3), as many researchers used in the past. The discrepancy is due to the assumptions made in the LSW theory. In the classical LSW theory, a near zero volume fraction was assumed for the dispersed phases within the matrix phase when coarsening starts, and the solute diffusion mainly occurs via bulk diffusion. However, this cannot be justified in real solidification and the effect of the volume fraction of solid must be considered [34]. If the volume fraction of the solid gradually increases, as in the cases of dendrite coarsening, interfacial diffusion become an important factor, consequently resulting in lower and lower coarsening rates, i.e., *n* > 3.

In our phase field governing equations, we assumed that there is no solute diffusion in the solid (*ϕ* = 1), but it is totally diffusive in the liquid (*ϕ* = −1). This means that the solute flux reaches its maximum value in the liquid. However, the vector of the solute concentration profiles (see Figure 10b_1_) clearly showed that the gradient of solute concentration (*c*/*c_∞_*) at *t* = 300 s mainly occurred at the solid-liquid interface and there was no obvious vector exit in the matrix liquid phase (see Figure 10b_2_,b_3_). The dominance of interfacial diffusion is obvious, and thus the exponent should be higher than 3. This argument has been supported by a number of previous studies [35,36,37,38], especially by the result from Terzi [29] (*n* = 4.4) and that of Poirier [30] (*n* = 4.5). Our experimental and phase field modeling works convincingly and showed that interfacial diffusion is a key mechanism in the coarsening of dendrites.

**Figure 11:**
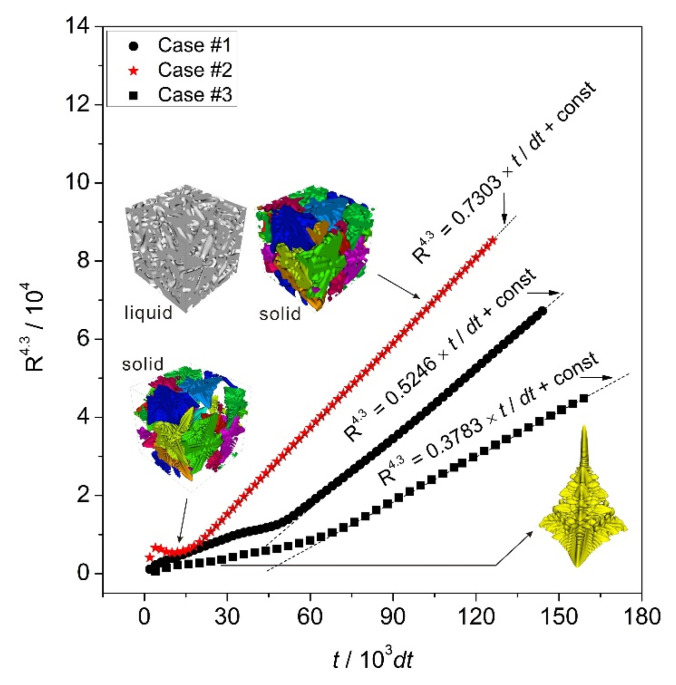
Comparison between simulation and fitted results for different simulation cases.

Simulations under different conditions were performed to test the validity of the fitted exponent *n* = 4.3 according to Equation (2). Three simulation cases were considered here. The key calculation parameters used for each case were the same as shown in Table 2, except that for case #1, *θ* = −0.12 and *ε*_1_ = 0.15 and for case #2, *θ* = −0.10 and *ε*_1_ = 0.15, while for case #3, *θ* = −0.12 and *ε*_1_ = 0.10. Here, the study was focused on the effect of undercooling and anisotropy strength. Besides, a lower scaling parameter, i.e., λ = 30, was employed due to the rather larger undercooling applied in the domain. For cases #1 and #2, the growth of multi-dendrites was simulated, while for case #3, only a single dendrite was considered. The corresponding simulation results are shown in Figure 11, in which the morphology of the dendritic microstructure at certain times is also shown. The characteristic length was fitted as a function of time in a manner similar to that used in Figure 10a.

As shown in Figure 11, the best fit always occurred when the exponent was set to be *n* = 4.3 regardless of the simulation cases. Comparing the results between case #1, lowering the temperature, i.e., increasing the supercooling, significantly facilitated the growth of the microstructure, but this change did not have any influence on the magnitude of the fitted exponent, and for both cases, *n* = 4.3. A similar situation occurred for case #2. By comparing case #3 to #1 and #2, it can be seen that the strength of the anisotropy did not influence the magnitude of the exponent either. These simulation results clearly indicated that dendritic coarsening followed the rule described by Equation (2), but with a different value of the exponent, i.e., *n* = 4.3 rather than *n* = 3, as predicted by the LSW theory.

## 6. Conclusions

In this paper, a 3D phase field model and real-time synchrotron X-ray tomography were used to study the dynamics of multi-dendrite concurrent growth and coarsening of an Al-15 *wt*.% Cu alloy. Based on the studies, the following conclusions can be drawn:

(1) Using the spatial locations and growth orientations of the real dendrites extracted from the tomography in the planted dendrite seeds at the start of the simulation, high fidelity multi-dendrite simulations have been achieved. The simulated and experimental dendrites showed high similarity in size, hierarchical dendrite branches, orientation, and surface morphology. This study has demonstrated that the phase field modeling is able to provide realistic results in the domain where experiments are very difficult or impossible to reach, such as in a very long time period of coarsening.
(2) The 3D datasets obtained from X-ray tomography experiments and phase field simulations reveal that, in a much more quantitative manner, the relative importance of the dendrite coarsening mechanisms at different stages of solidification. At higher temperatures, small arm melting (SAM) and interdendritic groove advancement (IGA) are the dominant mechanisms for dendritic coarsening, whereas at lower temperatures, i.e., when the solid volume fraction was higher than 0.70, the coalescence between neighboring arms became dominant. Meanwhile, the rate constant *k_c_* is highly dependent on the volume fraction of the solid (*f*s) and increases abruptly as *f*s approaches unity.
(3) Both phase field simulations and tomography experiments indicated that the coarsening of multi-dendrites indeed obeys the classical Lifshitz-Slyozov-Wagner theory [$R^{n}\;-{\;R}_{0}^{n}{\;=\;k}_{c}\left(t\;-t_{0}\right)$], but with the exponent *n* = 4.3. Phase field modeling indicated that this is mainly due to the effect of surface diffusion. This finding for dendrite coarsening has both scientific and technological significance.

## Figures and Tables

**Table 1:** The rotation of local dendrite axis to the global one.

Rotation Axis	Rotation Angle	Before Rotation	After Rotation	Location
*Y*	$\theta_{y}=\arctan\frac{u_{x}}{\sqrt{u_{y}^{2}{+u}_{z}^{2}}}$	*u*	$u_{1}{=R}_{y}{(\theta }_{y})u$	*YZ plane*
		*v*	$v_{1}{=R}_{y}{(\theta }_{y})v$	Unknown
*X*	$\theta_{x}=\arctan\frac{u_{1y}}{u_{1z}}$	$u_{1}$	$u_{2}{=-R}_{x}{(\theta }_{x}{)u}_{1}$	*Z axis*
		$v_{1}$	$v_{2}{=-R}_{x}{(\theta }_{x}{)v}_{1}$	Unknown
*Z*	$\theta_{z}=\arctan\frac{v_{2x}}{v_{2y}}$	$v_{3}$	$v_{3}{=-R}_{z}{(\theta }_{z}{)v}_{2}$	*XZ plane*

**Table 2:** The parameters used in the phase field simulation.

Parameter	Value
Liquidus slope, *m* (K/*wt*.%)	−3.4
Equilibrium partition coefficient, *k*	0.15
Anisotropy strength, *ε*_1_	0.06
Anisotropy strength, *ε*_2_	0.00
Gibbs-Thomson coefficient, *Γ* (K m)	2.4 × 10^−7^

## Data Availability

Data sharing not applicable.

## Supplementary Materials

The following are available online at https://www.mdpi.com/1996-1944/14/3/520/s1, Video S1. The growth and coarsening of Al-15 wt.% Cu alloy multiple dendrites, Video S2. The evolution of morphology and curvature for dendrite D1, Video S3. The evolution of morphology and curvature for dendrite D2, Video S4. Multi-dendrite growth and coarsening of subvolume V2 in Figure 2e, Video S5. Dynamic information of growth and coarsening for the simulated multiple dendrites.
